# A lover or a fighter? Opposing sexual selection pressures on men’s vocal pitch and facial hair

**DOI:** 10.1093/beheco/arv178

**Published:** 2015-11-01

**Authors:** Tamsin K. Saxton, Lauren L. Mackey, Kristofor McCarty, Nick Neave

**Affiliations:** Department of Psychology, Northumbria University, Northumberland Building, Ellison Place, Newcastle NE1 8ST, UK

**Keywords:** attraction, attractiveness, beards, dominance, facial hair, fundamental frequency, humans, intersexual selection, intrasexual selection, male–male competition, mate attraction, sexual selection, voice pitch.

## Abstract

The traditional assumption within the research literature on human sexually dimorphic traits has been that many sex differences have arisen from intersexual selection. More recently, however, there has been a shift toward the idea that many male features, including male lower-pitched voices and male beard growth, might have arisen predominantly through intrasexual selection: that is, to serve the purpose of male–male competition instead of mate attraction. In this study, using a unique set of video stimuli, we measured people’s perceptions of the dominance and attractiveness of men who differ both in terms of voice pitch (4 levels from lower to higher pitched) and beard growth (4 levels from clean shaven to a month’s hair growth). We found a nonlinear relationship between lower pitch and increased attractiveness; men’s vocal attractiveness peaked at around 96 Hz. Beard growth had equivocal effects on attractiveness judgments. In contrast, perceptions of men’s dominance simply increased with increasing masculinity (i.e., with lower-pitched voices and greater beard growth). Together, these results suggest that the optimal level of physical masculinity might differ depending on whether the outcome is social dominance or mate attraction. These dual selection pressures might maintain some of the documented variability in male physical and behavioral masculinity that we see today.

## Lay Summary

Men’s optimum masculinity depends on whether they want to attract partners or compete with rivals. We found that men’s voice pitch was most attractive around 1.5 standard deviations lower than average, whereas facial hair growth did not consistently affect attractiveness. In contrast, men were perceived ever more dominant with lower voices and more facial hair. Sexual selection consists of both attracting mates and competing against rivals, but here selection pressures might oppose each other somewhat.

## INTRODUCTION

Sexual selection comprises intersexual and intrasexual selection, relating respectively to choice by the opposite sex, and competition against members of the same sex. Reinforcing directional selection arises when intersexual and intrasexual selection pressures work together to promote a specific trait (e.g., when females choose males who compete successfully against other males, because this brings indirect benefits to the females). However, probably in rarer cases, intersexual and intrasexual selection can work antagonistically; females might not derive the most benefits from the most successful competitor if, for example, male investment in competition detracts from investment in offspring (see Hunt et al. 2009). In these cases, the optimum level of a trait might differ depending on whether we measure its success in intrasexual competition or mate attraction.

Humans are a useful species to choose for our current investigation of the interplay of intersexual and intrasexual selection pressures for 2 reasons. First, there has been a recent debate in the literature over whether men’s traits have arisen predominantly from intrasexual or intersexual selection (Puts 2010) that merits further exploration. Second, in humans, proxy measures of intersexual and intrasexual selection pressures have been developed and can be assessed relatively easily using rating tasks that assess preferences across a range of stimuli in a way that is not possible in other species. Intersexual selection is often inferred from women’s judgments of men’s physical attractiveness, which is a key component of human mate choice (see, e.g., Penton-Voak 2011). Intrasexual selection in contrast might be inferred based on ratings of men’s dominance (see, e.g., Barber 1995; Puts et al. 2006; Puts 2010; Puts et al. 2012), which might be used to manipulate access to potential partners (see, e.g., Hill et al. 2013).

Using these methods, there is evidence that opposing sexual selection pressures act on at least some men’s traits. Men perceived as most attractive are not those perceived as most dominant, and vice versa, when they are assessed based on their facial appearance (McArthur and Apatow 1984; Perrett et al. 1998; Johnston et al. 2001; Swaddle and Reierson 2002; Windhager et al. 2011; Stephen et al. 2012), facial hair growth (Neave and Shields 2008), or voice (Hodges-Simeon et al. 2010; Fraccaro et al. 2013). However, assessment of the individual traits does not tell us about the attractiveness or dominance of a trait in interaction with other traits. A previous study (Hill et al. 2013) found that the attractiveness of men’s facial and vocal masculinity interacted with their “girth” (a composite factor that represented their upper body measurements and weight). Men were perceived as more dominant at higher levels of girth, whereas women preferred men of intermediate girth. Yet higher levels of girth could still be attractive, so long as they were accompanied by lower levels of facial and vocal masculinity (Hill et al. 2013). This demonstrates that it is necessary to consider the multifaceted dimensions in which selection can act in order to understand trait selection pressures. In particular, a set of traits might have complementary or opposing effects on perceptions. For example, increasing the masculinity of 2 traits simultaneously might affect judges’ perceptions in ways that are different from increasing the masculinity of just one trait or the other; perceptual commonalities might have additive effects on judgments, whereas conflicting information might have interactive effects on judgments. In addition, the difference between optimum attractiveness and dominance might be clearest when the individual traits in question are perhaps most likely to arise from intrasexual than intersexual selection, such as men’s facial hair growth and lower-pitched voices, which might function to increase apparent size and dominance (Puts 2010). It is these 2 traits, in tandem, that the current study focused on.

Much evidence links voice pitch and facial hair growth to sexual selection. Both traits clearly distinguish men and women. Voices become lower pitched due to increasing mass and length of the vocal folds, which develop during puberty under the influence of testosterone (Hodges-Simeon et al. 2015). Hair growth, including facial hair growth, is dependent on androgens (Randall 2008). Consistent with a sexual signaling function, facial hair and men’s lower-pitched voices appear from puberty and have no obvious survival benefit. Facial hair is absent in women and children and is present to different extents in different human populations in ways that do not obviously relate to ecological or environmental variables (Barber 1995).

The current study set out firstly to investigate whether the optimum level of men’s facial hair growth and vocal pitch might differ depending on whether we assess attractiveness or dominance, that is, whether intrasexual selection pressures might be acting differently from intersexual selection pressures. Second, we wanted to investigate whether the 2 traits might interact in their effects on attractiveness ratings, such that, for example, higher levels of masculinity in one trait might offset lower levels in the other (cf. Hill et al. 2013). We used a unique, novel set of video stimuli to measure people’s perceptions of the dominance and attractiveness of men at different levels of voice pitch (4 levels, from low to high pitched) and beard growth (4 levels, from clean shaven to a month’s hair growth).

## METHODS

The study was given ethical approval under project reference RE-HLS-13-131025 by the Department of Psychology Ethics Committee in accordance with Northumbria University ethics and governance regulations.

### Stimuli

Six target men aged 19–21 were recruited from an opportunity sample of social contacts. Following time scales used in previous research on perceptions of facial hair growth (Dixson and Brooks 2013), the men visited the lab 4 times: once when clean shaven (the “clean shaven” condition), once 5 days after last shaving (“light stubble”), once 9–10 days after last shaving (“heavy stubble”), and once 4–6 weeks after last shaving (“beard”). The men wore a dark gown to cover any clothing and sat in front of a backdrop, facing a video camera, in a private room that was lit predominantly by artificial lighting in order to maintain constant lighting conditions across the different recordings. They were recorded using an Alesis AM1 microphone held around 10cm from their mouth as they said, “Hello, how are you?”, chosen as a familiar phrase that has relevance to interpersonal interactions (i.e., intrasexual and intersexual selection). The men were instructed to maintain a neutral facial expression during the recording. Recordings were made 5 times to allow participants to relax and to provide leeway for any technical issues. The use of video recordings in this experimental context is novel and allowed us to explore the effects of facial hair and voice manipulations in dynamic settings.

We also invited the men to return for a final recording session where they were recorded using a Digital Audio Workstation comprising a Saffire Pro 14 audio interface and an AKH C3000B condenser XLR microphone with stand and pop filter connected to an Apple MacBook Pro running sequencing software Avid Pro Tools. This final recording was used as the sound track that we manipulated (see below), and enabled us firstly to reduce the risk that the man’s voice could be influenced by his unaccustomed facial hair growth during the initial recording sessions and secondly to reduce to a minimum any possible artifacts or background noise that could be present in the voice recording. Two of the 6 men were not available for the final recording session, and so the recording from one of their original video sessions was used instead as the sound track that we manipulated. To confirm that these differences in recording conditions did not distinguish the stimuli, we also added the recording type as a between-subjects factor in the 2 repeated-measures analysis of the attractiveness ratings and the dominance ratings where the stimulus was unit of analysis (see below). There were no significant main effects of, or interactions with, the recording type (all *P*s > 0.17). In addition, the raters responded very similarly to each stimulus irrespective of which of the 2 types of voice recordings was used (see Results for details).

In order to create voice recordings that varied in fundamental frequency, these 6 sound files (one from each man) were manipulated in Praat 5.2.28 (Boersma 2001) by raising and lowering the fundamental frequency (*F*_0_) by 25 and 50 Hz, and thereby creating 4 new sound files from each man. Each sound file was then synchronized with and reattached to all of that man’s videos, creating a total of 96 videos (6 men × 4 sound files × 4 beard growth videos). We used a design that employed this range of phenotypes because this allows for the potential discovery of nonlinear sexual selection compared with designs that use dichotomous variables (Hunt et al. 2009). Prior to manipulation, the mean pitch in each recording from each man was 102, 106, 107, 124, 142, and 144 Hz, all of which fall within 1.5 standard deviations (SD) from a mean speaking fundamental frequency of adult men of around 120 Hz, with an SD of around 17 Hz (see, e.g., Nishio and Niimi 2008). Video editing was carried out in Windows Movie Maker. Videos were between 2 and 5s in length, with the majority around 3s in length; empirical evidence shows that this constitutes ample time for consistent social perceptions (Willis and Todorov 2006).

### Rating procedure

**Figure 2:**
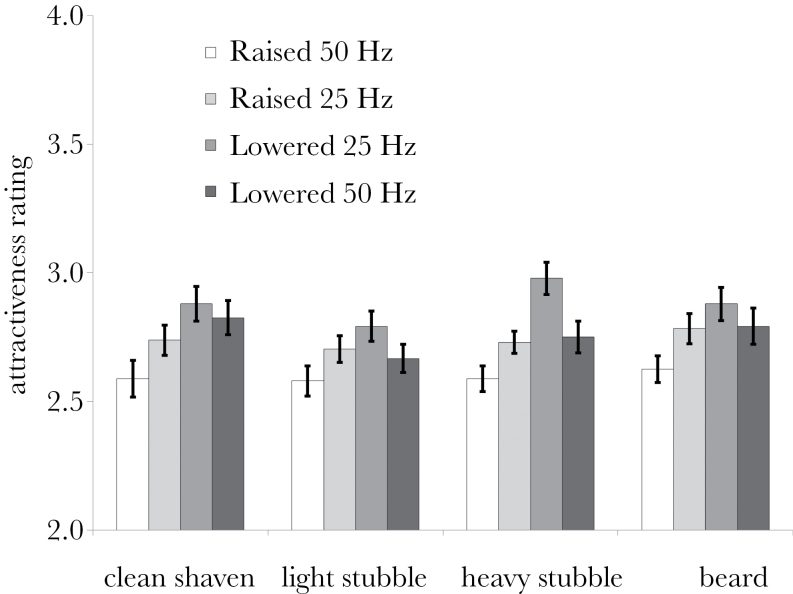
Estimated marginal means of attractiveness ratings of each of the voice pitch and facial hair combinations. Bars = mean ± standard error (calculated based on adjusted means; Loftus and Masson 1994; Field 2009).

Following sample size recommendations (Simmons et al. 2011), we recruited 40 raters (half male), aged 19–53 ($\bar{x}$ ± SD = 23±8 years) from an opportunity sample of social contacts. When we checked the analysis with the exclusion of the 3 participants aged >27 years, or the exclusion of the 5 participants aged >23 years, results were identical in their patterns of statistical significance, with the exception of some minor shifts in 6 (out of the 48 checked) of the significance value classifications given in Figure 2 (e.g., *P* < 0.001 changed to *P* < 0.01). The videos were presented to the raters in randomized order using the stimulus presentation package MediaLab (v2010, Empirisoft). For each video, participants had to use the keyboard to input their rating of the man’s attractiveness and dominance on 1–7 scales that used the anchors extremely unattractive and extremely attractive, and extremely submissive and extremely dominant. The attractiveness and dominance ratings were provided in separate blocks, and the order of the 2 blocks (attractiveness or dominance ratings provided first) was counterbalanced across raters.

### Analysis

The regression analyses were carried out in SAS v9.4 (Server Edition); the other analyses were carried out in SPSS v21. Greenhouse–Geisser correction was applied when Mauchly’s test indicated violations of the assumption of sphericity.

## RESULTS

Table 1 summarizes men’s and women’s dominance and attractiveness ratings of the 6 target men in the videos. Men gave slightly higher dominance ratings on average than women (Table 1), but the difference was not significant (*F*_1,39_ = 0.936, *P* = 0.339).

**Table 1:** Male and female raters’ ratings of the target men’s attractiveness and dominance

	Judgments of target men’s attractiveness				Judgments of target men’s dominance			
	Range	Mean	Kendall’s *W* to measure interrater agreement	*P*	Range	Mean	Kendall’s *W* to measure interrater agreement	*P*
Female raters (*n* = 20)	1–7	2.75	0.321	<0.001	1–7	2.86	0.179	<0.001
Male raters (*n* = 20)	1–7	2.74	0.197	<0.001	1–7	3.05	0.238	<0.001

We ran 2 repeated-measures Anovas with the raters as unit of analysis and rater sex as a between-subjects factor. The within-subjects factors constituted the 4 levels of facial hair growth, and the 4 levels of voice pitch. The dependent variables constituted the set of rating scores (either attractiveness or dominance) calculated from the average score given by a rater to all 6 men at each possible combination of facial hair growth and voice pitch.

**Figure 1:**
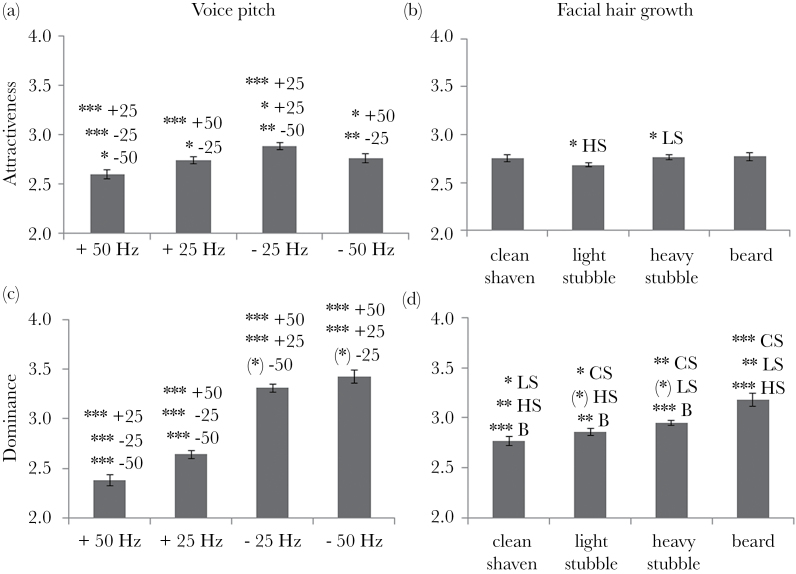
Estimated marginal means to show the effects of different levels of voice pitch (a and c) and facial hair (b and d) on ratings of attractiveness (a and b) and dominance (c and d). Text above each bar indicates where there is a significant difference in ratings compared with the CS (clean shaven), LS (light stubble), HS (heavy stubble), B (beard) facial conditions, or to the + (raised) or − (lowered) by 25 or 50 Hz voice pitch conditions, and at what significance level (least significant difference), where ***P < 0.001; **P < 0.01; *P < 0.05; (*) *P* < 0.09. Bars = mean ± standard error (calculated based on adjusted means; Loftus and Masson 1994; Field 2009). Effect sizes are given in Tables 2 and 3.

Ratings of dominance were affected by both voice pitch (*F*_1.5,55.2_ = 70.35, *P* < 0.001) and facial hair (*F*_1.4,54.0_ = 11.98, *P* < 0.001). As voice pitch lowered and facial hair increased, men were rated more dominant (Figure 1; Tables 2 and 3). There were no significant main effects of, or interactions with, rater sex (all *P* > 0.1). Examining each stimulus separately, dominance ratings increased consistently as voice pitch lowered for all except one man, where the mean dominance ratings of the lowest and second lowest pitches were reversed; as face hair increased, so mean dominance ratings increased (or in 2 contrasts remained constant) for 5 of the 6 men.

**Table 2:** Effect sizes (*r*) calculated from analysis of the effects of the different levels of voice pitch on ratings of attractiveness and dominance

	Attractiveness ratings			Dominance ratings		
	+25 Hz	−25 Hz	−50 Hz	+25 Hz	−25 Hz	−50 Hz
+50 Hz	0.55	0.51	0.31	0.69	0.86	0.82
+25 Hz		0.33	0.04		0.85	0.77
−25 Hz			0.46			0.29

**Table 3:** Effect sizes (*r*) calculated from analysis of the effects of the different levels of facial hair on ratings of attractiveness and dominance

	Attractiveness ratings			Dominance ratings		
	Light stubble	Heavy stubble	Beard	Light stubble	Heavy stubble	Beard
Clean shaven	0.26	0.01	0.03	0.34	0.41	0.54
Light stubble		0.33	0.22		0.28	0.50
Heavy stubble			0.03			0.55

Ratings of attractiveness were affected by voice pitch (*F*_1.5,58.3_ = 6.69, *P* = 0.005). Voices were perceived as most attractive when they were lowered by 25 Hz, compared with being lowered further by 50 Hz, or being raised (Figure 1; Table 2). The same pattern was apparent for each of the 6 stimuli: the voice recording lowered by 25 Hz always obtained a higher mean rating than any of the other 3 recordings of the same man, and the recording raised by 50 Hz was given the lowest mean rating for each man (or, in one instance, joint lowest). In contrast, there was no main effect of facial hair growth on ratings of attractiveness (*F*_2.1,80.1_ = 1.07, *P* = 0.352), and voice pitch and facial hair did not give rise to a significant interaction (*F*_6.1,232.7_ = 1.14, *P* = 0.339; Figure 2). There were no significant main effects of, or interactions with, rater sex (all *P* > 0.3).

In order to confirm whether the 2 types of ratings (attractiveness vs. dominance) were affected significantly differently by the facial hair compared to the voice pitch manipulations, we combined the 2 repeated-measures Anovas above into a single repeated-measures Anova that used the same variables, but included an additional within-subjects factor that specified whether the dependent variable was the attractiveness or dominance rating score. This confirmed that attractiveness and dominance ratings were affected significantly differently by the facial hair growth manipulation and by the voice pitch manipulation (type of rating × facial hair growth: *F*_1.6,62.3_ = 7.78, *P* < 0.001; type of rating × voice pitch: *F*_1.6,61.9_ = 35.1, *P* < 0.001).

For completeness, we also ran the analyses with the stimuli as unit of analysis. The pattern of results was identical. In a repeated-measures analysis with the 4 levels of facial hair and voice pitch as the within-subjects factors, voice pitch (*F*_3,15_ = 106.34, *P* < 0.001), and facial hair (*F*_3,15_ = 9.94, *P* = 0.001) both had a significant effect on dominance ratings, with no significant interaction (*F*_9,45_ = 1.22, *P* = 0.309). In the same analysis, but with attractiveness ratings instead of dominance ratings as the dependent variable, voice pitch had a significant effect on attractiveness ratings (*F*_1.3,6.25_ = 10.43, *P* = 0.014), but facial hair growth did not (*F*_3,15_ = 0.36, *P* = 0.780), and voice pitch and facial hair did not give rise to a significant interaction (*F*_3.6,18.16_ = 0.94, *P* = 0.456). Finally, combining these 2 repeated-measures Anovas into a single repeated-measures Anova that used the same variables, but also included an additional within-subjects factor that specified whether the dependent variable was the attractiveness or dominance rating score, again confirmed that attractiveness and dominance ratings were affected significantly differently by the facial hair growth manipulation and by the voice pitch manipulation (type of rating × facial hair growth: *F*_3,15_ = 5.49, *P* = 0.010; type of rating × voice pitch: *F*_3,15_ = 59.13, *P* < 0.001).

In order to calculate the most attractive voice pitch, we ran a nonlinear regression analysis using the proc-reg command in SAS. The outcome variable was the mean attractiveness rating given to each man at each of the 4 levels of voice pitch (averaging together ratings for all 4 levels of facial hair from all raters). The 6 men were dummy coded with the reference category set as the man whose mean attractiveness rating was closest to the sample mean. Voice pitch (i.e., the mean fundamental frequency of his original recording, ±25/50 Hz as appropriate) was entered as a linear and polynomial term. The overall model was significant (*F*_7,16_ = 103.79, *P* < 0.001, *R* ^2^ = 0.98), and so was the linear term (*P* = 0.0250) and the polynomial term (*P* = 0.0061). The most attractive pitch was at 96 Hz, which gained a mean attractiveness rating of 2.68 (β_0_ = 2.31380, pitch β = 0.00715, pitch^2^ β = −0.00003722). In a similar analysis, but with dominance ratings instead of attractiveness ratings as the outcome variable, and without the polynomial term, the equivalent parameter estimates were β_0_ = 4.50335, pitch β = −0.01100. Again, the overall model was significant (*F*_6,17_ = 59.30, *P* < 0.001, *R* ^2^ = 0.95), as was pitch (*P* < 0.0001); pitch as a polynomial term was not significant in the equivalent model (*P* = 0.8343).

## DISCUSSION

Our first aim was to determine whether optimum levels of voice pitch and facial hair growth differed depending on whether each was judged in an intersexual or intrasexual selection context (attractiveness or dominance). We found good evidence for this. There was a significant interaction between the type of rating (attractiveness or dominance) and the type of manipulation (voice pitch or facial hair growth). Although ratings of dominance increased linearly with increased facial hair growth or decreased voice pitch, attractiveness judgments were not obviously dependent on facial hair levels. The most attractive voices were those that were lowered by 25 Hz instead of 50 Hz, with an extrapolated maximum in attractiveness at 96 Hz.

That is, the optimum level of facial hair and voice pitch for male–male competition, assessed by ratings of dominance, differed significantly from the optimum level for mate attraction, assessed by ratings of attractiveness. The benefits that females derive from reproducing with the males that are the most dominant compared with most attractive might vary in time and space, leading to fluctuating selection pressures (Wong and Candolin 2005). Dominant individuals might enhance their reproductive success if their competitive strategies assist them in being better detected or more positively evaluated by potential choosers, or if their strategies lead to more mating opportunities (Wong and Candolin 2005). There are several examples within the human sexual selection literature of instances where the version of a trait or behavior that is rated most attractive by the other sex is different from the version believed to be most attractive to potential partners, including male muscularity (Pope et al. 2000), female make-up usage (Jones et al. 2014), and female adiposity (Fallon and Rozin 1985). Although this research is sometimes given as evidence that one sex misconstrues what the other sex wants, an alternative or complementary interpretation is that the differences arise from the differing requirements of successful inter- and intrasexual competition. Across species, opposing sexual selection pressures have been more commonly noted when mate attraction and mate competition occur sequentially instead of simultaneously (Hunt et al. 2009). This implies that manifestations of differences between inter- and intrasexual competitive behavior might differ most when comparing single- and mixed-sex groups. Indeed, in humans, adolescent aggression decreases when there is plenty of interaction between the sexes (Faris and Felmlee 2011).

Our findings of the effects of facial hair growth and voice pitch on dominance ratings are consistent with previous research. Men with facial hair are more likely to be perceived as more dominant (Pellegrini 1973; Addison 1989; Neave and Shields 2008), more masculine (Kenny and Fletcher 1973; Pellegrini 1973; Addison 1989; Dixson and Brooks 2013), more aggressive and strong (Addison 1989), and to have enhanced prosocial characteristics linked to social status, including social status itself (Dixson and Vasey 2012), self-confidence, courage and maturity (Pellegrini 1973), sincerity, enthusiasm, generosity, and extraversion (Kenny and Fletcher 1973), and enhanced parenting abilities and healthiness (Dixson and Brooks 2013). Men’s lower-pitched voices are rated as more dominant (Puts et al. 2006, 2007) and are perceived as belonging to larger, older, more masculine speakers (Collins 2000; Feinberg et al. 2005). Men lower the pitch of their voice in mating contexts when they believe themselves to be more dominant than their conversational partner (Puts et al. 2006); this artificial lowering may influence dominance rather than attractiveness or to a greater extent than attractiveness (Fraccaro et al. 2013).

Our findings on the attractiveness of slightly (but not excessively) lowered voice pitch are consistent with several studies that have raised and lowered voice pitch by 20 Hz or similar using an equivalent rectangular bandwidth manipulation and found that the lowered pitch was more attractive than the raised pitch (e.g., Feinberg et al. 2005, 2006, 2008; Vukovic et al. 2008; Saxton et al. 2009; Jones et al. 2010; Vukovic et al. 2010). Our findings are also consistent with a previous study that assessed the attractiveness of men’s voice pitch between 60 and 180 Hz and found that the most attractive voice pitch was approximately 96 Hz (Re et al. 2012); this value of 96 Hz is identical to the value identified in our study. Fundamental frequency varies with context, language, and age (see, e.g., Keating and Kuo 2012), but an attractiveness maximum at around 96 Hz is notably lower than the average male adult speaking fundamental frequency, which has a mean of around 120 Hz, and an SD of around 17 Hz (e.g., Nishio and Niimi 2008). It has been suggested previously (Re et al. 2012) that very low-pitched voices might be perceived unattractive because they indicate pathology, laryngeal damage caused by smoking, or overspending of resources in growing a larynx (e.g., hyperpituitarism). Although these reasons could explain the lack of appeal of very low-pitched voices, they do not explain why the lowest pitches used in our study were perceived as more dominant but less attractive than slightly higher pitches. However, high levels of masculinity have been linked to lower levels of relationship stability (Booth and Dabbs 1993; Mazur and Michalek 1998) and less investment in relationships and parenting (Booth and Dabbs 1993; Perrett et al. 1998; Gangestad and Simpson 2000; Wynne-Edwards 2001); these explanations are consistent with the finding that very low-pitched men’s voices are perceived as unattractive but dominant.

In the current study, we did not find that any facial hair level was clearly more attractive than all the rest. Previous research variously describes the attractiveness of a lack of facial hair (Cunningham et al. 1990; Wogalter and Hosie 1991; Muscarella and Cunningham 1996; Dixson and Vasey 2012; Geniole and McCormick 2015); the attractiveness of increased facial hair (Pellegrini 1973; Reed and Blunk 1990; Hellström and Tekle 1994); and the attractiveness of an intermediate level of facial hair (Feinman and Gill 1977; Neave and Shields 2008; Dixson et al. 2013; Dixson and Brooks 2013). Some of the differences between the studies probably arise from differences in the stimuli. Previous studies have variously used photographs, computer-generated images, line drawings, and written descriptions; the studies also vary in their choice of facial hair density, length and distribution, and the number of different categories of facial hair growth tested. A second contributor to the differences likely arises from variables that can change significantly between local cultures, samples, populations, and across time (see, e.g., Dixson et al. 2013; Dixson and Vasey 2012; Dixson and Brooks 2013). Preferences for greater levels of facial hair growth fluctuate (Robinson 1976), and have been linked to the rarity value of facial hair (Janif et al. 2014), and the marriage market (Barber 2001) among other things. Women’s preferences for male masculinity have been linked to many diverse within- and between-subject variables, including menstrual cycle stage (Jones et al. 2008), testosterone (Welling et al. 2007), relationship status (Little et al. 2002), age (Saxton et al. 2009; Little et al. 2010), menopause status (Jones et al. 2011), etc. We chose not to control for these variables so that our raters represent a typical sample of their demography (i.e., younger adults, socioeconomic status, and local culture consistent with social groups surrounding a university in the north-east of England, etc.), and as such are most generalizable to the average preferences of that group. As is standard within psychology studies, the results might not be generalizable to populations that differ widely from that sampled. Nor did we distinguish attractiveness for short- versus long-term relationships or measure other individual differences that might shape preferences for masculinity compared with femininity (see, e.g., Puts, Jones et al. 2012), due to the exigencies of focusing on one research question at a time.

Why should masculinization of voice be more clearly linked than masculinization of facial hair to differences in attractiveness judgments? The difference might arise because of the differences in the biological significance of lowered voice pitch compared with facial hair growth. The relationship between testosterone level and voice fundamental frequency seems relatively robust (Dabbs and Mallinger 1999; Evans et al. 2008), and voice fundamental frequency has been linked to the condition and biological quality of the individual (Puts, Apicella et al. 2012; Hodges-Simeon et al. 2014, 2015). In contrast, there might be only a very slight relationship between individual differences in androgen levels and facial hair growth. The establishment of male pattern hair growth, including facial hair growth, relies on androgens (see, e.g., Randall et al. 1991), but once established at puberty, some aspects of male facial hair growth can be sustained even in the absence of male levels of circulating androgens (Giltay and Gooren 2000). Although one study (Farthing et al. 1982) found a link between testosterone levels and hair density and linear hair growth, this was obtained in only a small sample (*n* = 20) that collapsed together 2 groups (celiacs and healthy controls), which differed in facial hair prevalence and androgen levels. A larger sample, albeit in a different population (!Kung San and Kavango men from Namibia/Southern Africa), found that sex hormones in serum and saliva were linked to various patterns of bodily hair growth but not to facial hair growth (Winkler and Christiansen 1993). Although there is likely a small correlation between androgen levels and hirsutism in women (e.g., Reingold and Rosenfield 1987; Mueller et al. 2007; Legro et al. 2010), it is not clear that this relationship should automatically translate to men. It might be that relationships between androgens and facial hair growth have not been uncovered because simple measurements of androgen levels in bodily fluids such as saliva are probably overly simplistic when hair growth depends on the intensity of androgen action in the target cells, which is part of a much bigger physiological dynamic that also depends on the sensitivity of those cells (Ebling 1986; Winkler and Christiansen 1993; Zitzmann and Nieschlag 2001). However, overall, there is not strong evidence that individual differences in the extent of facial hair growth are good indices of individual androgen levels (see, e.g., Ebling 1986), and any link may be reduced still further because facial hair is easily groomed to comply with local cultural conventions.

A second aim of the study was to determine whether the optimum levels of voice pitch and facial hair interacted with each other, such that less masculinity in one trait could offset more masculinity in the other. We found no evidence for this; attractiveness judgments were not affected by a significant interaction between voice pitch and facial hair. Previous research has provided evidence of such an interaction in relation to men’s body size and facial masculinity (Hill et al. 2013) but not in relation to men’s facial and vocal masculinity (O’Connor et al. 2012). It is possible that the interaction would become apparent, but only with further masculinization of facial hair or voice pitch, or a larger sample of stimuli or raters. Although our stimuli were limited in number, this is in line with some other studies on facial hair (Reed and Blunk 1990; Muscarella and Cunningham 1996; Neave and Shields 2008). The pattern of our results was identical when we ran the analyses with the stimuli as unit of analysis, suggesting that the results should be generalizable beyond the stimuli used. However, because features of the stimuli might interact with the manipulations, replication in a larger sample of stimuli would be useful. Unlike computer-generated or -manipulated images that can hold all other variables constant, one limitation of using real men is that their own perceptions of themselves might vary with their facial hair growth, for example, by increasing or decreasing confidence, and so add confounding variables to their behavior that shapes attractiveness and/or dominance ratings (cf. Wood 1986). However, there is value in complementing the studies that have used computer-generated images with those using real-life facial hair growth, and the present study is the first to do so using video recordings of men at different facial hair levels. Together, these results suggest that the optimal level of physical masculinity may differ depending on whether the aim is social dominance or mate attraction. Indeed, there is much evidence that investment in intrasexual competition is traded-off against investment in paternal investment (see, e.g., Heath and Hadley 1998; Geary 2000), perhaps supported by changes in testosterone levels (Wingfield et al. 1990). Such trade-offs are contingent on differences in mating systems (Dixson et al. 2005) and ecological factors (Grueter et al. 2015). These dual selection pressures might maintain some of the documented variability in male physical and behavioral masculinity that we see today.

## Funding

This work was supported by a research bursary from the Department of Psychology, Northumbria University, to L.L.M.
